# Automated Insulin Delivery in Pregnancy With Type 1 Diabetes: An Integrative Review of Clinical Evidence, Socio‐Technical Implementation and Equity

**DOI:** 10.1155/jdr/1989683

**Published:** 2026-09-22

**Authors:** Jieli Li, Tina Nan Qi, Xiangmin Tan, Rasika Jayasekara

**Affiliations:** ^1^ Faculty of Medicine, Nursing and Health Sciences, Monash University, Melbourne, Victoria, Australia, monash.edu.my; ^2^ Faculty of Medicine, Dentistry and Health Sciences, The University of Melbourne, Melbourne, Victoria, Australia, unimelb.edu.au; ^3^ Nursing and Midwifery, College of Sport, Health and Engineering, Victoria University, Melbourne, Victoria, Australia, vu.edu.au; ^4^ College of Health, School of Nursing and Midwifery, Adelaide University, Adelaide, South Australia, Australia, adelaide.edu.au

**Keywords:** automated insulin delivery, diabetes education, health equity, hybrid closed-loop, implementation, pregnancy, type 1 diabetes

## Abstract

Automated insulin delivery (AID) has moved rapidly from experimental use to emerging clinical implementation in pregnancy complicated by type 1 diabetes. Randomised trials now show that selected systems can improve pregnancy‐specific continuous glucose monitoring outcomes, particularly time in range and overnight glycaemic stability, but the evidence is system‐specific rather than class‐wide and remains underpowered for definitive maternal and neonatal endpoints. This integrative review synthesises clinical trial, observational, qualitative, implementation, policy and equity evidence to examine how AID can be translated into safe, equitable and sustainable perinatal diabetes care. We argue that AID in pregnancy is best understood as a socio‐technical intervention: its effectiveness depends on the interaction between algorithm design, pregnancy physiology, user‐device behaviours, education, workforce capability, antenatal workflows, delivery planning, postpartum dose reduction and access to specialist support. We summarise system‐specific evidence for CamAPS FX, MiniMed 780G and Tandem Control‐IQ, review gaps in maternal and neonatal outcome data and propose a practical implementation framework spanning patient, device, clinician, service and health‐system levels. The review also presents a reproductive‐stage care pathway from preconception to postpartum and identifies equity risks across high‐income and lower‐resource settings. The field has moved beyond asking whether selected AID systems improve glucose metrics; the next responsibility is to determine how services can deliver these benefits safely, affordably and equitably for the pregnant people most likely to benefit.

**Key points**

• Selected AID systems improve pregnancy‐specific glycaemic exposure, but the evidence is system‐specific rather than a generic class effect.
• Maternal and neonatal endpoints remain insufficiently powered; current evidence supports glycaemic benefit and reassuring safety, not definitive perinatal outcome reduction.
• AID in pregnancy should be implemented as a socio‐technical intervention requiring device‐specific education, workforce training, service pathways and equity safeguards.

## 1. Introduction

Pregnancy complicated by type 1 diabetes requires unusually tight and dynamic glycaemic management. Insulin resistance rises across gestation, insulin requirements change rapidly, nausea and altered intake increase hypoglycaemia risk, and postprandial glucose excursions have direct relevance for fetal growth. Continuous glucose monitoring (CGM) established that pregnancy‐specific glucose exposure is clinically meaningful, with the CONCEPTT trial showing improvement in maternal glycaemia and neonatal outcomes when CGM was used in type 1 diabetes pregnancy [1]. AID therefore enters a context in which the clinical need is clear but the implementation environment is complex.

Recent randomised trials have changed the evidence base. AiDAPT demonstrated substantial improvement in pregnancy‐specific time in range with CamAPS FX [2]; CRISTAL showed that MiniMed 780G improved overnight glycaemia and reduced hypoglycaemia but did not significantly improve overall pregnancy time in range [3]; and CIRCUIT demonstrated clinically meaningful improvement in pregnancy‐specific time in range with Tandem Control‐IQ [4]. These trials make two points simultaneously. First, AID can improve glycaemic exposure in type 1 diabetes pregnancy. Second, this effect is not a generic closed‐loop class effect. Device design, target flexibility, pregnancy‐specific algorithm behaviour, user input, baseline glycaemia and service support all matter. Additional assisted hybrid closed‐loop and earlier research‐grade pregnancy studies have provided supporting evidence on feasibility, glycaemic effects and implementation constraints [5–7].

Several recent qualitative, implementation, review, expert‐guidance and consensus publications have addressed patient perspectives, healthcare‐professional experiences, clinical efficacy, safety and practical use of diabetes technologies in pregnancy [8–14]. This review therefore does not seek to replicate another broad efficacy review. Instead, it addresses the remaining translational question: how can health services implement specific AID systems safely, equitably and sustainably across preconception, pregnancy, delivery and postpartum care? The aim is to integrate system‐specific clinical evidence with qualitative, implementation, workforce, policy and equity evidence, and to translate that evidence into a socio‐technical implementation framework for clinical services.

## 2. Methods

### 2.1. Review Design

We conducted an integrative review to allow synthesis across heterogeneous evidence types, including randomised trials, observational studies, qualitative and mixed‐methods studies, implementation reports, international consensus recommendations, regulatory documents and health‐system policy sources. This design was selected because the review question concerns clinical effectiveness and translation into service delivery, rather than efficacy alone.

### 2.2. Evidence Sources and Eligibility

Clinical evidence was drawn from studies evaluating AID or hybrid closed‐loop systems in pregnancy complicated by type 1 diabetes, including randomised controlled trials, crossover trials, comparative cohorts and single‐arm observational studies [2–7]. Implementation and experience evidence included qualitative and mixed‐methods studies of pregnant users and healthcare professionals, together with expert guidance on off‐label or pregnancy‐specific use [9–14]. Policy and equity evidence included consensus statements, national guidance, regulatory decisions and reimbursement or access documents relevant to AID in pregnancy [8, 15–25]. Evidence was prioritised when it reported system‐specific outcomes, pregnancy‐relevant CGM targets, maternal or neonatal endpoints, safety outcomes, user experience, workforce requirements or access barriers.

Because this review was designed as an integrative review rather than a PRISMA‐style systematic review, evidence was selected to support transparent synthesis across clinical, experiential, implementation, policy and equity domains. We prioritised peer‐reviewed trials and comparative studies for system‐specific clinical evidence; qualitative and mixed‐methods studies for user and healthcare‐professional experience; consensus and expert guidance for pregnancy‐specific clinical recommendations; and regulatory, reimbursement and policy documents for implementation and equity analysis. We did not conduct formal risk‐of‐bias assessment or duplicate independent screening because the purpose was not to generate a new pooled efficacy estimate, but to integrate heterogeneous evidence into a service‐facing implementation framework.

### 2.3. Synthesis Approach

Evidence was synthesised narratively and thematically. Clinical evidence was organised by AID system and outcome domain. Maternal, neonatal and safety outcomes were interpreted cautiously because existing studies were not primarily powered for these endpoints. Qualitative and implementation evidence was mapped to a socio‐technical framework comprising five interacting levels: patient and family; device and algorithm; clinician and workforce; service pathway; and health‐system and equity context. This framework was then used to derive practical implementation strategies and a staged care pathway from preconception to postpartum.

### 2.4. Relationship to Related Systematic Review Work

This integrative review is distinct from a conventional systematic review or meta‐analysis. Quantitative estimates from trial and observational evidence are used to contextualise the implementation problem, but the purpose of this article is to integrate clinical, experiential and policy evidence into a service‐facing framework. The focus is therefore on what the evidence requires clinical services to do, rather than on producing a new pooled estimate of efficacy.

## 3. System‐Specific Clinical Evidence

The contemporary evidence requires system‐specific interpretation. CamAPS FX, MiniMed 780G and Tandem Control‐IQ differ in algorithm design, target flexibility, pregnancy suitability, evidence base, regulatory status and practical support requirements. Table 1 summarises the evidence most relevant to clinical implementation.

**Table 1:** System‐specific evidence and implementation implications for AID in type 1 diabetes pregnancy.

System	Key pregnancy evidence	Glycaemic effect	Maternal/neonatal and safety signal	Pregnancy‐specific strengths	Implementation limitations
CamAPS FX	AiDAPT randomised trial in pregnancy complicated by type 1 diabetes; international consensus and regulatory discussions now place this system at the centre of pregnancy‐specific AID evidence [2, 8, 17].	Pregnancy‐specific TIR was 68.2% with closed‐loop versus 55.6% with standard care; adjusted mean difference 10.5 percentage points (95% CI 7.0 to 14.0), approximately 2.5 additional hours/day [2]. Overnight TIR also improved.	No clear increase in severe hypoglycaemia or diabetic ketoacidosis in the major trial context. Maternal and neonatal endpoints remain underpowered for definitive conclusions.	Adjustable lower glucose targets are more compatible with pregnancy than many non‐pregnancy‐specific systems; strongest pregnancy‐specific evidence among contemporary commercial systems.	Requires compatible pump/CGM components, trained clinical teams, patient education about modes such as Boost/Ease‐Off, and sustained antenatal and postpartum support. Access varies by jurisdiction and component availability.
MiniMed 780G	CRISTAL randomised trial compared MiniMed 780G with standard insulin therapy in specialist centres in Belgium and the Netherlands [3].	Overall pregnancy TIR averaged across gestation was 66.5% with AHCL versus 63.2% with standard care; adjusted mean difference 1.88 percentage points (95% CI ‐0.82 to 4.58). Overnight TIR, time below range and treatment satisfaction improved [3].	No unexpected safety signal; severe events were uncommon. Clinical endpoints remain insufficiently powered.	Widely used AHCL platform; useful signal for overnight stability and hypoglycaemia reduction.	Lowest target and algorithm behaviour may not fully match pregnancy targets. Postprandial control remains dependent on user behaviour, pre‐bolus timing, carbohydrate estimation and clinician‐guided setting adjustment.
Tandem t:slim X2 with Control‐IQ/Control‐IQ+	CIRCUIT multicentre randomised trial assessed Control‐IQ during pregnancy with type 1 diabetes; subsequent regulatory expansion has increased relevance for clinical practice [4, 18].	Pregnancy‐specific TIR from 16 to 34 weeks was 65.4% with closed‐loop versus 50.3% with standard care plus CGM; adjusted mean difference 12.5 percentage points (95% CI 9.5 to 15.6), approximately 3 additional hours/day [4].	Safety data are reassuring in the trial context, but maternal and neonatal endpoints are not definitive.	Adds contemporary RCT evidence beyond CamAPS FX and supports broader but still system‐specific AID benefit.	Original algorithm was not designed specifically for pregnancy targets. Access may be constrained by insurance coverage, pump warranty, regulatory status, device compatibility and local staff expertise.
Earlier research‐grade or experimental closed‐loop systems	Early Cambridge and model‐predictive‐control studies demonstrated feasibility and provided mechanistic support for pregnancy‐specific closed‐loop development [6, 7, 11].	Short‐term and crossover studies generally supported improved overnight control and feasibility, but were small and not designed for contemporary implementation questions.	Insufficiently powered for perinatal outcomes; useful primarily as proof‐of‐concept and feasibility evidence.	Showed that pregnancy‐oriented algorithms and intensive support models are technically achievable.	Limited direct applicability to current commercial systems; implementation lessons must be translated cautiously.

## 4. Maternal, Neonatal and Safety Outcomes

A central implementation issue is that the most reproducible benefit of AID in pregnancy is improvement in CGM metrics rather than proven reduction in hard pregnancy outcomes. Improved time in range is meaningful and plausibly linked to foetal exposure, but current trials and cohorts are not large enough to establish definitive reductions in outcomes such as large‐for‐gestational‐age birth, neonatal hypoglycaemia, preterm birth, pre‐eclampsia or neonatal intensive care admission [2–5]. Table 2 summarises outcome domains that should be presented quantitatively but interpreted cautiously.

**Table 2:** Maternal, neonatal and safety outcome signals from comparative AID pregnancy evidence.

Outcome domain	Quantitative signal from comparative evidence	Interpretation for implementation
Pregnancy‐specific time in range	Overall pooled mean difference approximately +10.40 percentage points (95% CI 9.20 to 11.59). Trimester‐specific improvements were also reported in the systematic review dataset: first trimester +5.11%, second trimester +6.14%, third trimester +6.16%.	Supports AID as a glycaemic optimisation tool when the system, settings and clinical support are appropriate.
Overnight glycaemia	Overnight TIR increased by approximately +14.55 percentage points (95% CI 9.20 to 19.90); overnight hyperglycaemia decreased by approximately −11.95 percentage points; overnight TBR <63 mg/dL decreased by approximately −1.10 percentage points.	Overnight benefit is a strong value proposition for women and services, particularly where fear of nocturnal hypoglycaemia and sleep disruption are prominent.
Pre‐eclampsia	Pooled OR 1.05 (95% CI 0.60 to 1.83); I^2^ approximately 14%.	Do not claim obstetric risk reduction. Continue standard obstetric risk assessment and surveillance.
Gestational weight gain	Pooled mean difference −0.58 kg (95% CI −2.51 to 1.35); heterogeneity substantial.	Insufficient evidence for a weight‐gain effect; interpretation should remain cautious.
Preterm delivery	Pooled OR 0.76 (95% CI 0.52 to 1.12); I^2^ approximately 0%.	Perinatal endpoint‐powered studies are needed before guideline claims can move beyond glycaemic benefit.
Large‐for‐gestational‐age/macrosomia	Pooled LGA OR 0.98 (95% CI 0.73 to 1.32); I^2^ approximately 0%.	AID may reduce glycaemic exposure, but foetal growth is multifactorial and requires integrated obstetric surveillance.
Small‐for‐gestational‐age	Pooled OR 0.73 (95% CI 0.47 to 1.13); I^2^ approximately 0%.	No definitive growth‐safety signal; continue monitoring foetal growth according to standard pregnancy diabetes pathways.
Neonatal hypoglycaemia requiring glucose treatment	Pooled OR 0.79 (95% CI 0.56 to 1.10); I^2^ approximately 0%.	Use as a key future endpoint; avoid implying that TIR gains automatically translate into neonatal outcome gains.
NICU admission	Pooled OR 0.93 (95% CI 0.64 to 1.34); I^2^ approximately 0%.	No definitive reduction established; neonatal care outcomes require larger or registry‐linked datasets.
Stillbirth or neonatal death	Pooled OR 0.75 (95% CI 0.18 to 3.22).	Events are rare and estimates are imprecise; this outcome cannot be interpreted as evidence of benefit or harm.
Diabetic ketoacidosis	Pooled OR 0.77 (95% CI 0.18 to 3.24).	Implementation must include sick‐day rules, ketone education and infusion‐set failure pathways.
Severe hypoglycaemia	Pooled OR 1.15 (95% CI 0.58 to 2.28).	No clear increased safety signal, but wide confidence intervals require cautious counselling and postpartum hypoglycaemia prevention.

## 5. AID as a Socio‐Technical Intervention

AID is often described as a device intervention, but in pregnancy, it functions as a socio‐technical intervention. The algorithm cannot be separated from pregnancy physiology, user‐device behaviours, clinical interpretation of data, local training capacity, obstetric workflows and access pathways. This reframing is necessary because the same device may perform differently depending on gestational stage, baseline glycaemia, meal bolus behaviour, target settings, troubleshooting support and clinician experience [9–14].

Qualitative studies show that women may experience reduced anxiety, improved sleep, greater confidence and a sense of normality, but they may also face alarms, connectivity problems, device visibility, adhesion issues, obsessive data checking, difficulty trusting the algorithm and the emotional burden of trying to protect the baby [9, 11–13]. Healthcare professional studies similarly show enthusiasm for AID, but also emphasise workforce training, mentorship, download interpretation, local protocols and the challenge of maintaining expertise when relatively small numbers of pregnant women use these systems [10, 14].

Table 3 translates this evidence into a clinical‐service framework. The purpose is not to imply that every service can offer AID immediately; rather, it identifies the interacting components that must be in place when AID is used or considered.

**Table 3:** Socio‐technical implementation framework for AID in type 1 diabetes pregnancy.

Level	Key barriers	Facilitators	Implementation strategies	Clinical service owner
Patient/user	Alarm burden; CGM lag; device visibility; adhesion problems; anxiety/guilt; variable trust; missed or delayed meal boluses; carbohydrate counting difficulty.	Perceived safety; improved sleep; discreet bolusing; reduced fear of hypoglycaemia; improved confidence after training.	Structured onboarding; teach‐back; pregnancy‐specific pre‐bolus coaching; troubleshooting scripts; psychosocial screening; partner/family support where appropriate.	Diabetes educator, diabetes specialist nurse, dietitian, midwife.
Device/algorithm	Target mismatch with pregnancy goals; variable target flexibility; postprandial excursions; closed‐loop exits; infusion‐set failure; sensor/pump compatibility.	Systems with lower target options; clear modes for temporary adjustment; reliable downloads; remote monitoring.	Device‐specific protocols; settings review schedule; escalation triggers; ketone and infusion‐set failure pathway; postpartum target reset.	Endocrinologist, diabetes educator, device‐trained nurse.
Clinician/workforce	Uneven pump/AID expertise; limited exposure to pregnant users; difficulty interpreting downloads; unclear responsibility across professions.	Mentorship, case review meetings, checklists, super‐user models, multidisciplinary clinics.	Competency‐based training; system‐specific checklists; shared documentation; case‐based supervision; protected time for data review.	Diabetes service lead, nursing/midwifery education lead.
Service pathway	Fragmented preconception, antenatal, delivery and postpartum workflows; unclear intrapartum plan; poor handover after birth.	Integrated diabetes‐obstetric clinics; remote monitoring; planned postpartum dose reduction; clear delivery protocol.	Stage‐specific care pathway; preconception referral triggers; antenatal review cadence; delivery plan; breastfeeding and postpartum hypoglycaemia plan.	Multidisciplinary diabetes‐in‐pregnancy team.
Health system/equity	Cost; insurance or subsidy limits; pump warranty restrictions; rurality; language barriers; digital literacy; limited specialist services; LMIC insulin/monitoring constraints.	National reimbursement; public CGM/pump funding; telehealth; culturally safe education; prioritisation criteria.	Equity audit; subsidy navigation; translated resources; rural telehealth models; workforce outreach; technology‐access ladder from insulin and CGM to AID.	Health service, funder, policy‐maker.

## 6. A Staged Care Pathway for Clinical Services

**Figure 1:**
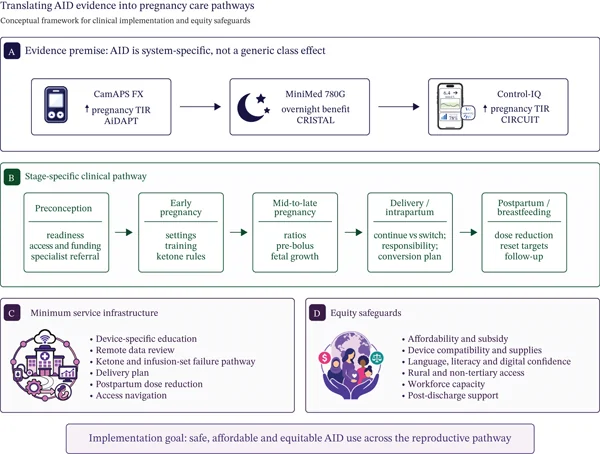
Translating AID evidence into pregnancy care pathways. The framework links system‐specific AID pregnancy evidence with stage‐specific clinical implementation, minimum service infrastructure and equity safeguards. Panel (A) highlights that current AID pregnancy evidence is system‐specific rather than a generic class effect. Panel (B) summarises the reproductive‐stage care pathway from preconception to postpartum. Panel (C) identifies the minimum service infrastructure required for safe implementation. Panel (D) outlines equity safeguards needed to support access beyond highly resourced specialist settings. AID, automated insulin delivery; CGM, continuous glucose monitoring; TIR, time in range.

The implementation framework can be operationalised as a reproductive‐stage care pathway. The pathway should not imply that AID is appropriate or available for every woman in every setting; rather, it identifies the minimum clinical processes required when AID is used or considered. Figure 1 provides a service‐facing conceptual framework that links system‐specific AID evidence with the staged reproductive care pathway, minimum service infrastructure and equity safeguards; the key pathway actions are expanded in Table 4.

**Table 4:** Operational detail for the proposed AID pregnancy care pathway.

Stage	Core clinical questions	AID‐specific actions	Equity check
Preconception	Is pregnancy planned? Is glycaemia optimised? Is the woman already using CGM/pump/AID?	Assess technology readiness; explain pregnancy targets and device limitations; review funding/subsidy/insurance; establish referral to specialist diabetes‐in‐pregnancy care.	Does the woman have affordable access to CGM, pump supplies, compatible devices and specialist support?
Early pregnancy	Can AID be initiated or optimised safely before major insulin resistance develops?	Confirm settings; provide device‐specific training; manage nausea/vomiting and hypoglycaemia; set ketone and infusion‐set failure rules; schedule frequent data review.	Are language, literacy, rurality, work/caring demands or cost limiting engagement?
Mid‐to‐late pregnancy	Are rising insulin requirements and postprandial excursions being managed?	Review insulin‐to‐carbohydrate ratios, pre‐bolus timing, correction strategy, site changes, sensor reliability and foetal growth context.	Can the service provide intensified support without privileging only those with high digital confidence or flexible work schedules?
Delivery/intrapartum	Can AID safely continue, or should local insulin protocol be used?	Document criteria for continuing AID; identify who reviews glucose/device data; prepare conversion to IV/subcutaneous insulin if needed.	Does the birth setting have staff familiar with the chosen device and escalation plan?
Postpartum/breastfeeding	How will insulin be reduced rapidly and safely?	Implement immediate dose reduction; reset targets as appropriate; prevent hypoglycaemia during breastfeeding; arrange follow‐up and technology debrief.	Is postpartum support accessible after discharge, including for women outside tertiary centres?

## 7. Nursing, Midwifery and Diabetes Education Implications

The revised implementation argument gives nursing, midwifery and diabetes education a central rather than peripheral role. In trials and routine care, AID benefit depends on repeated education, data interpretation, anticipatory guidance, troubleshooting and continuity across pregnancy stages [9, 10, 14]. Diabetes educators and specialist nurses often translate algorithm behaviour into daily practices: earlier meal bolusing, carbohydrate estimation, infusion‐set management, recognising closed‐loop exits, interpreting nocturnal patterns and responding to persistent hyperglycaemia. Midwives and obstetric teams are essential for integrating device decisions with foetal growth, delivery planning, breastfeeding and postpartum safety. The service model must therefore treat workforce preparation as part of the intervention, not as an afterthought. A clinically usable AID pathway should specify which team member is responsible for onboarding, data review, troubleshooting, intrapartum handover, postpartum insulin reduction and post‐discharge follow‐up.

## 8. Equity and Global Implementation

Equity is now one of the defining implementation challenges. In high‐income settings, the barrier is often not simple regulatory absence but fragmented access: device approval may differ by jurisdiction, pumps and CGM may be funded through different schemes, insurance or pump warranty rules may limit switching systems during pregnancy, and local services may lack staff trained in pregnancy‐specific AID support [4, 15–20]. In Australia, CGM access for pregnancy may be more supported than pump and AID access, creating a practical gap between monitoring and automated delivery [19, 20]. In the United Kingdom, national implementation of hybrid closed‐loop therapy recognises pregnancy and pregnancy planning, but rollout remains dependent on trained specialist services [15, 16]. In the United States, regulatory clearances are expanding, yet access is still mediated by insurance coverage, device compatibility and service capacity [17, 18].

The global equity question is sharper in lower‐resource settings. In many parts of Africa, Southeast Asia and other low‐ and middle‐income regions, reliable insulin access, glucose monitoring, antenatal diabetes care, trained workforce and referral pathways may be more immediate priorities than AID deployment [21–24]. AID should therefore be framed as one level in a technology‐access continuum: uninterrupted insulin and antenatal care everywhere, self‐monitoring and CGM where feasible, and AID where the required education, supplies, clinical support and safety pathways can be sustained. An equity‐oriented implementation framework should avoid creating a two‐tiered future in which the highest‐risk women are least likely to access the technologies and services that could reduce glycaemic burden.

Cost‐effectiveness evidence should also be interpreted through this implementation lens. An economic evaluation of the CRISTAL trial suggested that MiniMed 780G AHCL could be cost‐saving from a Belgian healthcare‐payer perspective when shorter and less frequent diabetes‐related admissions offset device costs, although the authors emphasised the need for more robust data on pregnancy and longer‐term outcomes [25]. Future cost‐effectiveness analyses should therefore compare not only devices, but models of care, including training time, data‐review workload, avoided admissions, neonatal intensive care use, maternal quality of life, travel burden and the opportunity cost of specialist workforce capacity.

## 9. Research and Policy Agenda

Recent clinical standards, practical obstetric guidance, real‐world Control‐IQ experience and postpartum follow‐up data further emphasise that future work should extend beyond antepartum glucose metrics to include intrapartum and postpartum safety, service workflows, patient experience and sustained access [26–29].

• Comparative‐effectiveness studies that evaluate systems separately rather than pooling AID as a single class.
• Trials and registry‐linked analyses powered for maternal and neonatal endpoints, including LGA/macrosomia, neonatal hypoglycaemia, preterm birth, NICU admission and pre‐eclampsia.
• Standardised reporting of pregnancy CGM metrics, overnight outcomes, postprandial exposure, closed‐loop use, user behaviours and clinical contact intensity.
• Implementation trials comparing models of education, remote monitoring, nurse‐led follow‐up, midwifery integration and postpartum transition support.
• Equity‐focused research reporting ethnicity, socioeconomic status, rurality, language, health literacy, insurance/subsidy status and baseline technology access.
• Cost‐effectiveness analyses that include device costs, training time, remote‐monitoring workload, avoided complications, maternal quality of life and downstream neonatal care costs.

## 10. Conclusion

AID has created a new standard of possibility in type 1 diabetes pregnancy. Selected systems can provide clinically meaningful improvements in pregnancy‐specific glycaemic exposure, especially overnight, without a clear signal of increased serious harm. However, the evidence is system‐specific, implementation‐dependent and not yet definitive for maternal and neonatal clinical outcomes. The next phase should therefore be judged not only by better algorithms, but by whether services can deliver AID safely, affordably and equitably across preconception, pregnancy, delivery and postpartum care. This requires a socio‐technical approach in which women, devices, clinicians, care pathways and health systems are understood as interacting components of the intervention.

## Author Contributions

Jieli Li: conceptualisation, methodology, investigation, resources, writing – original draft, writing – review and editing, visualisation, and project administration. Tina Nan Qi: conceptualisation, methodology, investigation, writing – review and editing. Xiangmin Tan: writing – review and editing. Rasika Jayasekara: supervision and writing – review and editing.

## Funding

No funding was received for this manuscript.

## Disclosure

All authors have read and approved the final version of the manuscript. Jieli Li, as corresponding author and manuscript guarantor, takes responsibility for the integrity and accuracy of the review synthesis. Journal articles cited in this manuscript were checked against PubMed where indexed and by title search against Retraction Watch/Retraction Database web results on 24 August 2026. No cited journal article was identified as retracted during these checks. Published corrections were reviewed where visible and none was judged to alter the interpretation of the citation for this review.

## Ethics Statement

Ethical approval was not required because this integrative review synthesised published literature, guidelines, regulatory documents and policy sources only, and did not involve recruitment of human participants, collection of primary data, access to identifiable records, or intervention with participants.

## Conflicts of Interest

The authors declare no conflicts of interest.

## Data Availability

The authors confirm that all data supporting the findings of this study are available within the article and its cited sources. No new primary datasets were generated or analysed.
